# Aortic Leaflet Stresses Are Substantially Lower Using Pulmonary Visceral Pleura Than Pericardial Tissue

**DOI:** 10.3389/fbioe.2022.869095

**Published:** 2022-04-26

**Authors:** Ye Chen, Xiao Lu, Haoxiang Luo, Ghassan S. Kassab

**Affiliations:** ^1^ California Medical Innovations Institute, San Diego, CA, United States; ^2^ Department of Mechanical Engineering, Vanderbilt University, Nashville, TN, United States

**Keywords:** aortic valve, cardiovascular flow, fluid–structure interaction, leaflet stresses, tissue stiffness

## Abstract

**Background:** Porcine heart and bovine pericardium valves, which are collagen-based with relatively little elastin, have been broadly utilized to construct bioprosthetic heart valves (BHVs). With a larger proportion of elastin, the pulmonary visceral pleura (PVP) has greater elasticity and could potentially serve as an advantageous biomaterial for the construction/repair of BHVs. The question of how the aortic valve’s performance is affected by its bending rigidity has not been well studied.

**Methods:** Based on the stress–strain relationships of the pericardium and PVP determined by planar uni-axial tests, a three-dimensional (3D) computational fluid–structure interaction (FSI) framework is employed to numerically investigate the aortic valve’s performance by considering three different cases with Young’s modulus as follows: E=375 , 750 , and 1500 kPa, respectively.

**Results:** The stroke volumes are 112, 99.6, and 91.4 ml as Young’s modulus increases from 375 to 750 and 1500 kPa, respectively. Peak geometric opening area (GOA) values are 2.3, 2.2, and 2.0 cm^2^ for E=375 , 750, and 1500 kPa, respectively. The maximum value of the aortic leaflet stress is about 271 kPa for E=375 kPa, and it increases to about 383 and 540 kPa for E=750 and 1500 kPa in the belly region at the peak systole, while it reduces from 550 kPa to 450 and 400 kPa for E=375 , 750, and 1500 kPa, respectively, at the instant of peak “water-hammer”.

**Conclusion:** A more compliant PVP aortic leaflet valve with a smaller Young’s modulus, E , has a higher cardiac output, larger GOA, and lower hemodynamic resistance. Most importantly, the aortic leaflet stresses are substantially lower in the belly region within the higher compliance PVP aortic valve tissue during the systole phase, even though some stress increase is also found during the fast-closing phase due to the “water-hammer” effect similar to that in the pericardial tissue. Future clinical studies will be conducted to test the hypothesis that the PVP-based valve leaflets with higher compliance will have lower fatigue or calcification rates due to the overall lower stress.

## Introduction

Aortic valves may be subject to thickening and calcification, which causes aortic valve stenosis and cardiac dysfunction. Multiple bioprosthetic valves, such as the bovine pericardium and porcine aortic valve, have been FDA-approved for clinical use to replace the dysfunctional cusps in patients (Manji et al., 2012; Kheradvar et al., 2015; Dasi et al., 2017). Calcification of bioprosthetic heart valves in patients is still a significant clinical problem, although various improvements have been suggested for mitigation. The magnitude of mechanical stress is known as a risk factor for the calcification of leaflets (Ferrans et al., 1978; Thubrikar et al., 1983; Robicsek and Thubrikar, 2002; Schoen and Levy, 2005; Singh et al., 2008). Calcification of bioprosthetic heart valves in recipient patients causes deterioration of valvular function and eventually requires reoperation. It is reported that calcification in bioprostheses begins in the areas of greatest mechanical stress (Ferrans et al., 1978; Thubrikar et al., 1983; Robicsek and Thubrikar, 2002; Schoen and Levy, 2005; Singh et al., 2008). The significant mechanical stress in bioprostheses of the porcine heart valve and bovine pericardium damages collagen fibers and/or disrupts collagen structural integrity by the sliding of individual layers of collagen over each other, i.e., mechanical stresses initiate calcification by damaging the structural integrity of the leaflet tissue (Ferrans et al., 1978; Thubrikar et al., 1983). Calcification of bioprostheses can be inhibited by reducing functional stresses through the modification of design and tissue properties (Thubrikar et al., 1983). Therefore, the simulation of mechanical stresses in heart prosthetic valves is fundamental to heart valve design and longevity.

Due to recent advances in computational modeling algorithms and high-performance computing techniques, the computational fluid–structure interaction (FSI) has become a standard and affordable tool for the investigation and evaluation of heart valve performance, i.e., there have been substantial computational efforts devoted to the study of the interaction between the heart valve and blood flow (Griffith et al., 2009; Borazjani, 2013; Gilmanov et al., 2015; Hsu et al., 2015; Kamensky et al., 2015; Gilmanov and Sotiropoulos, 2016; Gilmanov et al., 2019; SoltanySadrabadi et al., 2021). FSI simulations of the heart valves are capable to address several substantial challenges previously encountered, including the large three-dimensional (3D) deformation of the valve, topological change of the flow domain due to the valve’s opening and closure, numerical instability of the FSI algorithm, and high computation expense, either relying on the immersed boundary (IB) type of the approach (Griffith et al., 2009; Borazjani, 2013; Gilmanov et al., 2015; Gilmanov and Sotiropoulos, 2016; Gilmanov et al., 2019; SoltanySadrabadi et al., 2021) or those using the boundary-conformal mesh (Hsu et al., 2015; Kamensky et al., 2015). By introducing normalized bending rigidity $E_{B}^{*}$ , we have demonstrated the optimal range of $E_{B}^{*}\;$ between 0.003 and 0.04 for the proper aortic valve opening area. Excessively smaller or larger $E_{B}^{*}$ will either give rise to severe leaflet fluttering or incur difficulty of the aortic valve’s full opening (Chen and Luo, 2018; Chen and Luo, 2020). Despite these successful FSI computational studies on the heart valves opening area, hemodynamics, and deformation pattern, the correlation of the stresses within the leaflet tissue with the bending rigidity remains unexplored, even though recent FE analysis suggested that the regions with significantly elevated mechanical stress are strongly correlated with the regions with a high risk of calcium buildup (Qin et al., 2020). From the viewpoint of solid mechanics, leaflets with a smaller Young’s modulus correspond to lower stresses in the tissue if the deformations are similar, which may be instrumental in reducing fatigue and calcification of the prosthetic valve.

Recently, we evaluated the bovine/porcine pulmonary visceral pleura (PVP) as a potential biomaterial for prosthetic valves, where the elastic modulus is one order magnitude smaller than that of the bovine pericardium and porcine aortic valve (Lu et al., 2020). In this work, we used the same 3D FSI approach as described in our previous work (Chen and Luo, 2018; Chen and Luo, 2020) to investigate the effect of tissue stiffness and bending rigidity on the aortic valve’s performance by the selection of three different Young’s moduli: E=375 , 750 , and 1500 kPa, while keeping the leaflet thickness uniform at 0.3 mm. The modulus E=375 kPa corresponds to the stress–strain relationship of the more compliant PVP as determined by planar uni-axial tests, while E=1500 kPa represents that of stiffer porcine heart valves and the bovine pericardium. We computed the flow rate, geometric opening area (GOA), leaflet deformation, hemodynamic resistance, and mechanical stresses of the aortic valve. In particular, the aortic leaflet stresses and their potential connection with calcified aortic valve disease (CAVD) are discussed.

## Model Setup and Numerical Approach

### Model Setup

**FIGURE 1:**
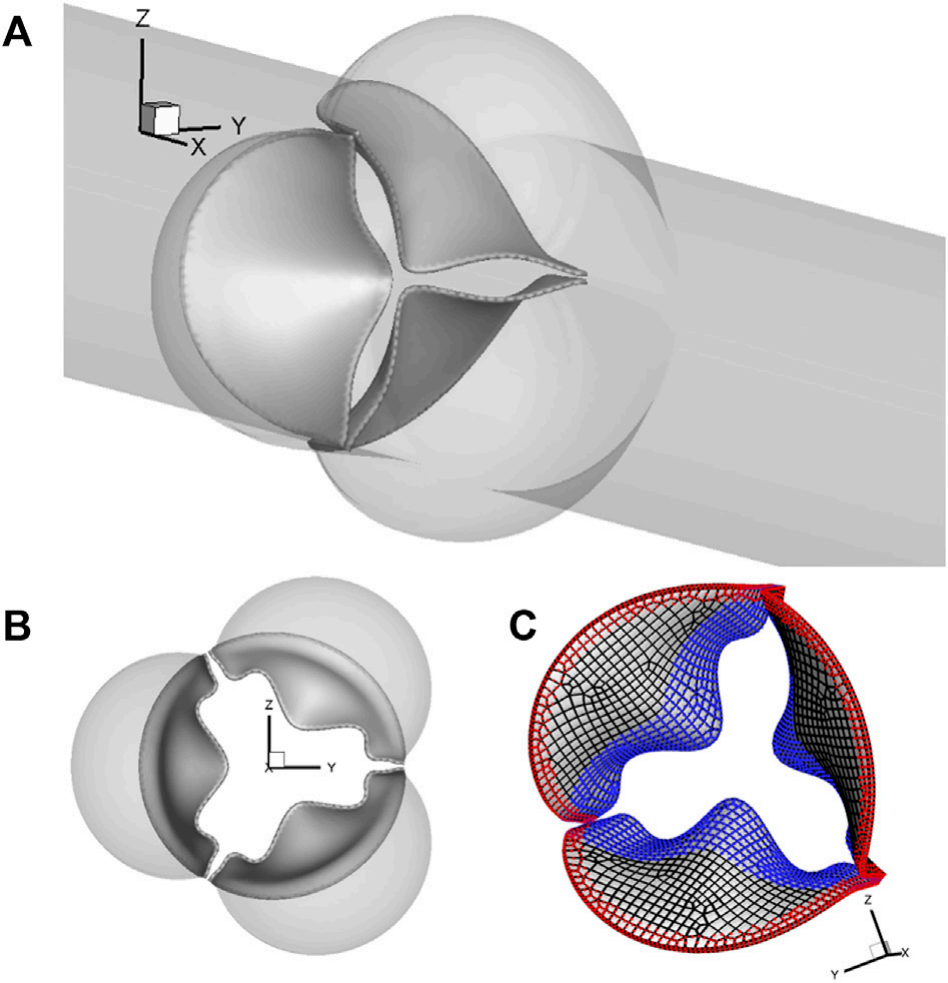
**(A)** Computational model of the aorta root, where the aortic valve is placed within the sinus region of the simplified straight aorta. **(B)** Aortic valve and three sinuses. **(C)** Fixed nodes (red markers) and the prescribed contact detection region (blue markers).

**FIGURE 2:**
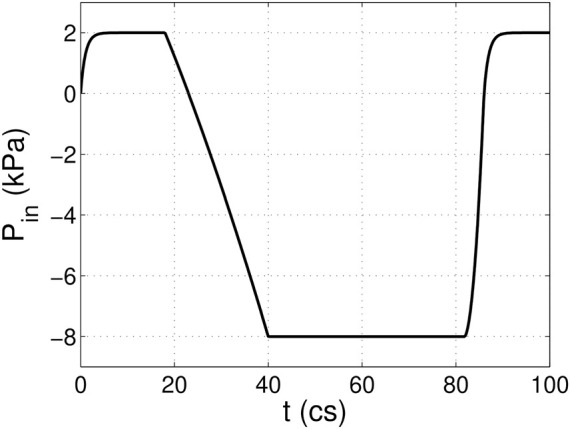
Pressure load applied at the inlet of the aorta tube in the FSI simulation, where cs denotes centiseconds.

A 3D computational model similar to that in our previous work (Chen and Luo, 2018; Chen and Luo, 2020) is adopted and shown in Figure 1. The aorta is simplified to a cylindrical tube of diameter D = 2.1 cm and length L = 19 cm. It includes three-lobed dilation to represent the aortic sinuses, of which the geometry and dimensions are based on physiological measurements of the human aortic root (Swanson and Clark, 1974; Reul et al., 1990). A tri-leaflet aortic valve is positioned within the sinus region, and its three flexible leaflets can deform independently from each other. Despite the variability of the anatomy of the human aorta, simplified computational domains similar to this are often used for the FSI study of the native aortic valve and its prostheses (Borazjani, 2013; Hsu et al., 2015; Kamensky et al., 2015; Griffith, 2012; Marom et al., 2013; Mao et al., 2016). Similar to previous FSI studies (Borazjani, 2013; Kamensky et al., 2015), a transient transvalvular pressure load is applied at the inlet of the aorta tube to drive the blood flow (see Figure 2). The exit pressure at the outlet is 0 kPa. This pressure drop is consistent with previous studies of the human aorta (Hole, 1996; Kim et al., 2008). Since the aortic wall is assumed to be rigid in the model, which is a limitation to our study, the specific reference pressure at the outlet does not matter, and the FSI is purely driven by the pressure difference between the two ends.

For spatial discretization, the aorta wall is divided into 20,735 triangular surface elements with local refinement in the sinus region, where the element size is about 0.3 mm. A uniform thickness of 0.3 mm is assumed for each leaflet, which consists of a total of 539 FE serendipity (20-node Hexahedron) elements and 4,021 nodes. The meshes of the aorta wall and leaflets are separate, and they intersect each other without necessarily sharing the nodes. The aorta wall is assumed to be rigid, while the leaflets can undergo free deformations. The discretization of 20,735 triangular surface elements of the aorta wall serves as a physical boundary in the immersed boundary method–based simulation, where the no-slip and no-penetration conditions are applied when solving the Navier–Stokes equations. On each leaflet, 830 nodes (red markers) located in both the commissure region of two neighboring leaflets and along the base are fixed, while 903 nodes (blue markers) consisting of the prescribed contact region are used to prevent leaflet inter-penetration, as shown in Figure 1C.

Aortic valves are known to be nonhomogeneous (Kuang et al., 2017; Oveissi et al., 2020), and different constitutive models have been proposed to investigate the mechanical behavior and the failure mechanisms of the aortic valve (Weinberg and Kaazempur-Mofrad, 2005; Mao et al., 2016). In this work, the hyperelastic Saint Venant–Kirchhoff model is adopted to represent the tissue behavior of the valvular leaflets. The constitutive relationship of the Saint Venant–Kirchhoff model can be expressed as follows:

$$\sigma_{ij}^{K}=D_{ijmn}E_{mn}$$ (1)

where $D_{ijmn}$ is the elastic matrix with 12 non-zero elements that depend on Young’s modulus E and Poisson’s ratio $\nu_{s}$ , and $E_{mn}$ is the Lagrangian strain tensor (Tian et al., 2014).

**FIGURE 3:**
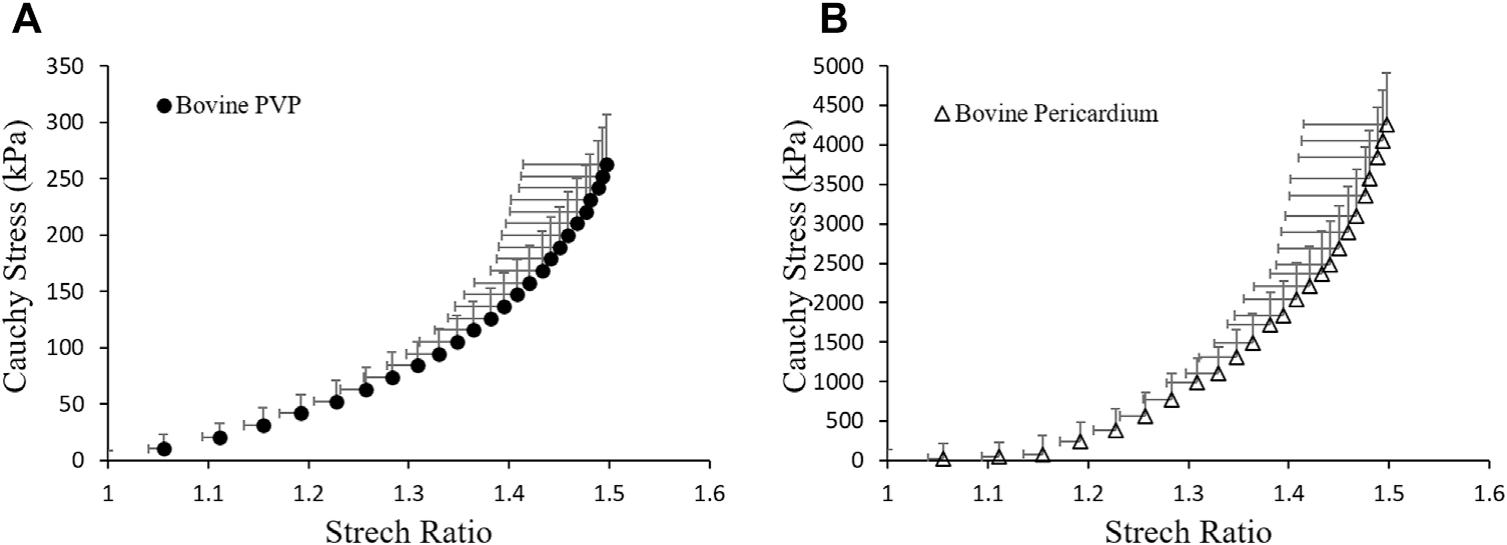
Material properties of **(A)** bovine PVP and **(B)** bovine pericardium measured from planar uni-axial tests.

The leaflet dynamics is governed by the following equation:

$$\rho_{s}\frac{d^{2}u_{i}}{dt^{2}}+\eta_{d}\frac{du_{i}}{dt}=\frac{\partial \sigma_{ij}}{\partial x_{j}}$$ (2)

where $u_{i}$ is the nodal displacement, $\eta_{d}$ is the damping coefficient representing structural damping of the tissue, and $\sigma_{ij}$ is the Cauchy stress tensor. The density of the leaflets is $\rho_{s}$ = 1 g/cm^3^, and $\eta_{d}$ is chosen to be 100 g/cm^3^·cs to ensure the reasonable time scale for valve opening and closing (Chen and Luo, 2018; Chen and Luo, 2020). Three different values of Young’s modulus, E=375 , 750 , and 1500 kPa are selected to represent the difference in the stress–strain relationship between the more compliant PVP and the stiffer pericardium measured from planar uni-axial tests (see Figure 3). The Poisson’s ratio $\nu_{s}$ = 0.4 is used for all three cases (Hsu et al., 2015; Kamensky et al., 2015).

The blood is assumed to be Newtonian and incompressible. The governing equation of the flow is the unsteady Navier–Stokes equation as follows:

$$\frac{\partial v_{i}}{\partial x_{i}}=0$$ (3)

$$\rho \left(\frac{\partial v_{i}}{\partial t}+\frac{\partial v_{i}v_{j}}{\partial x_{j}}\right)=-\frac{\partial p}{\partial x_{i}}+\mu \frac{\partial^{2}v_{i}}{\partial x_{j}\partial x_{j}}$$ (4)

where $v_{i}$ is the velocity component, p is the pressure, ρ is the blood density, and μ is the dynamic viscosity. No-slip and no-penetration boundary conditions are imposed on the aorta wall and the leaflet surface. The fluid domain is a 19 × 4.4 × 4.4 cm^3^ rectangular bounding box and is divided by a 400 × 130 × 130 non-uniform Cartesian grid. A fine resolution with ∆x = 0.025 cm and ∆y = ∆z = 0.034 cm is used in the region around the aortic valve. A mesh refinement study has been conducted to validate the accuracy of the current mesh, in which the flow domain is divided by a 500 × 260 × 260 non-uniform Cartesian grid with a resolution around the leaflets of ∆x = 0.015 cm and ∆y = ∆z = 0.017 cm, and the mesh for the finite element model of the valve is increased by approximately five times. Comparisons of the leaflet dynamics, hemodynamic resistance of the aortic valve, transient flow rate, and pressure distribution on the leaflet surface have shown that the results between the baseline and the refined mesh are in excellent agreement (Chen and Luo, 2020). The density and dynamic viscosity of the blood are ρ = 1 g/cm^3^ and μ = 0.005 Pa s, respectively (Chen and Luo, 2018; Chen and Luo, 2020).

### Numerical Approach

We applied an in-house computational approach that was previously developed for simulating biological systems involving large deformations to solve the heart valve FSI problem (Luo et al., 2012; Tian et al., 2014). In this approach, the flow and solid solvers are arranged in a partitioned manner such that the flow is simulated using an accurate direct-forcing IB method based on a Cartesian grid, and the solid is solved using a nonlinear FE method. Strong FSI coupling is achieved by iterating the two solvers while communicating the transient position and velocity information at the fluid–structure interface until convergence. Detailed explanations of the numerical algorithms and their parallel implementation are included in our previous work (Chen and Luo, 2018; Chen and Luo, 2020). Each cardiac cycle has a duration of T = 0.86 s, which corresponds to a heart rate of 70 beats per minute. For convenience, we used centisecond (cs) as the time unit thereafter. To ensure numerical stability of the FSI coupling, the time step used for the flow solver is ∆t = 4.0 × 10^–3^ cs. The time step for the structural simulation is smaller, ∆t = 5.0 × 10^–5^ cs, so that each FSI step contains 80 sub-steps for the solid.

To accelerate the costly FSI simulation, a parallel computing technique based on a domain decomposition strategy has been implemented on the flow side. On the structure side, multiple OpenMP threads are forked on a single CPU processor, and the data communication between the fluid and solid sides is achieved *via* message passing interface (MPI) calls. Details of the implementation have been discussed in our previous work (Chen and Luo, 2018; Chen and Luo, 2020). For the FSI simulations in this study, the rectangular flow computation domain is divided into 13 subdomains in each of the y- and z-directions, which requests a total of 169 CPU cores. A separate CPU core handles the computation of solid mechanics, and the FE solid solver is parallelized using 16 OpenMP threads. Each heart valve FSI simulation takes approximately 50  h for one cardiac cycle on Stampede 2 partitions at the Texas Advanced Computing Center (TACC).

The contact force was calculated to prevent the inter-penetration between leaflets, especially during the closing phase. At each time step, the contact distance d of the nodes within the prescribed contact detection region (blue markers in Figure 1C) was first calculated by projecting it onto the surface of its neighboring leaflets. $d_{0}\;$ is a prescribed threshold of distance. When $d<d_{0}$ , the contact algorithm is activated, and the contact force is calculated as follows:

$$f_{cnt}=-f_{ext}-k\left(d-d_{0}\right)$$ (5)

where $f_{cnt}$ is the nodal contact force, $f_{ext}$ is the hydrodynamic force from the blood flow on the node, and k is the contact stiffness. It should be noted that the external load $f_{ext}\;$ is canceled out, and a net force of magnitude $-k\left(d-d_{0}\right)$ is added to prevent inter-penetration of colliding leaflets when $d<d_{0}$ . It is noted that the contact force vanishes outside of the contact distance (when $d>d_{0}$ ). Here, we set k=0.04 g/cs^2^ and $d_{0}=0.08$ cm so that the leaflets are stopped without collision, and the gap between them is below one fluid cell width (Chen and Luo, 2018; Chen and Luo, 2020).

## Results

### Flow Rate and Valve Opening Area

**FIGURE 4:**
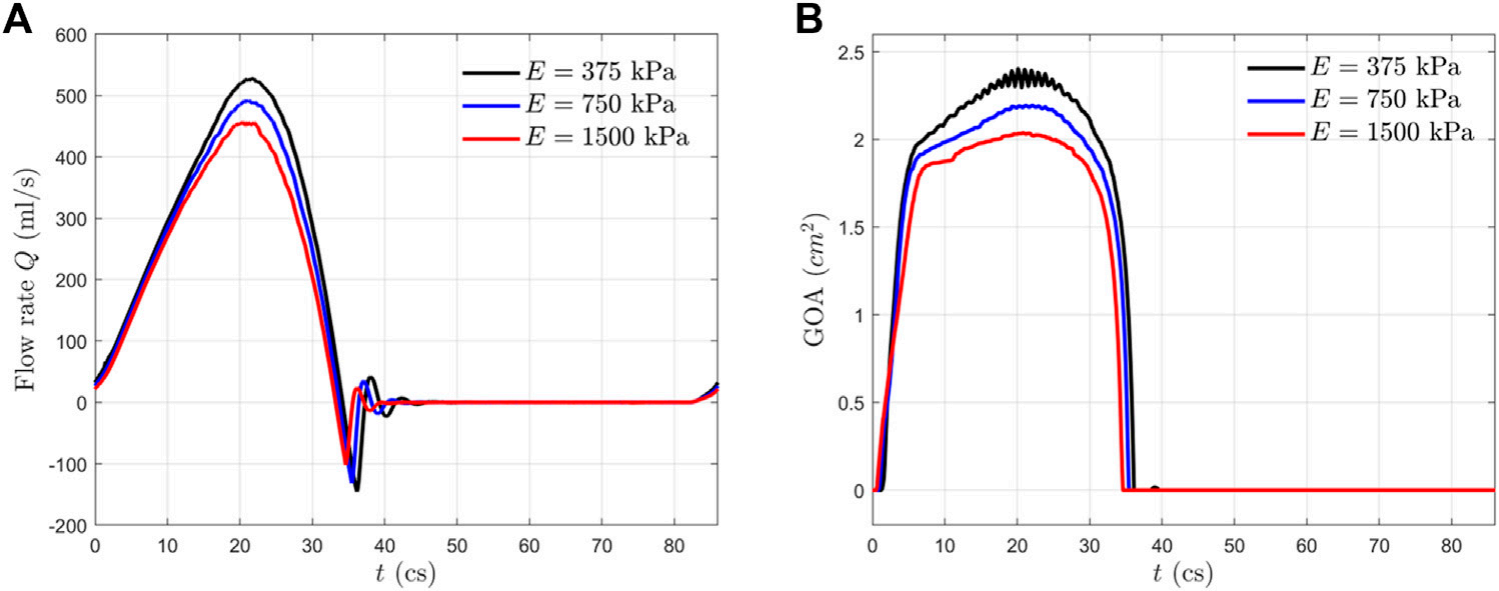
**(A)** Transient flow rate Q and **(B)** GOA for the three FSI cases.

In Figure 4, we presented the transient flow rate Q and valve geometric opening area (GOA) for the three FSI cases. The flow rate is calculated at the outlet. The peak flow rate reduces from 527 and 492 to 459 ml/s as Young’s modulus increases from 375 to 750 and 1500 kPa. Integrating the transient flow rate in time, we obtained the corresponding stroke volume, and they were 112 , 99.6 , and 91.4  ml, respectively, which are within the physiological range for normal adults (Griffith et al., 2009; Borazjani, 2013). During the valves’ rapid closure, the negative dip flow rates were −144 , −131 , and −102 ml/s for E=375 , 750 , and 1500 kPa, respectively. Valve reverberation during rapid closure corresponds to the clinical second heart sound (S2) and has an indication of heart function. Stronger reverberation is found for the more compliant aortic leaflet valve. We also calculated the regurgitation volume, which represents the blood leakage backward through the aortic valve toward the inlet during diastole. For E=375 , 750 , and 1500 kPa, the regurgitation volume is less than 0.3 ml per cardiac cycle.

The GOA from the FSI simulation is presented in Figure 4B for all three cases. This area is calculated by projecting the valve in the axial direction and finding the opening area. From the GOA history, we observed that all the aortic valves opened rapidly in 5 to 6 cs and experienced rapid closure within 3 to 4 cs. The more compliant aortic valve with a smaller E=375  kPa opened slightly faster while shutting down slightly delayed by scrutinizing the GOA profile. Also, a larger GOA value after reaching full open is observed for the more compliant leaflets. The peak GOA values were about 2.3 , 2.2  , and 2.0  cm^2^ for E=375 , 750  and 1500 kPa, respectively, at the peak systole. In addition, a small oscillation is observed for the more compliant valve ( E=375 kPa) during the peak systole. This oscillation is related to the fluttering motion of the leaflets’ free edge after it reached the fully open stage, and it is negligible when compared with the more compliant aortic leaflet valves with thicknesses of 0.05, 0.08, and 0.1 mm, as shown in Ref. 16. All three valves closed properly without leakage since their GOAs reached zero during diastole.

### Leaflet Deformation

**FIGURE 5:**
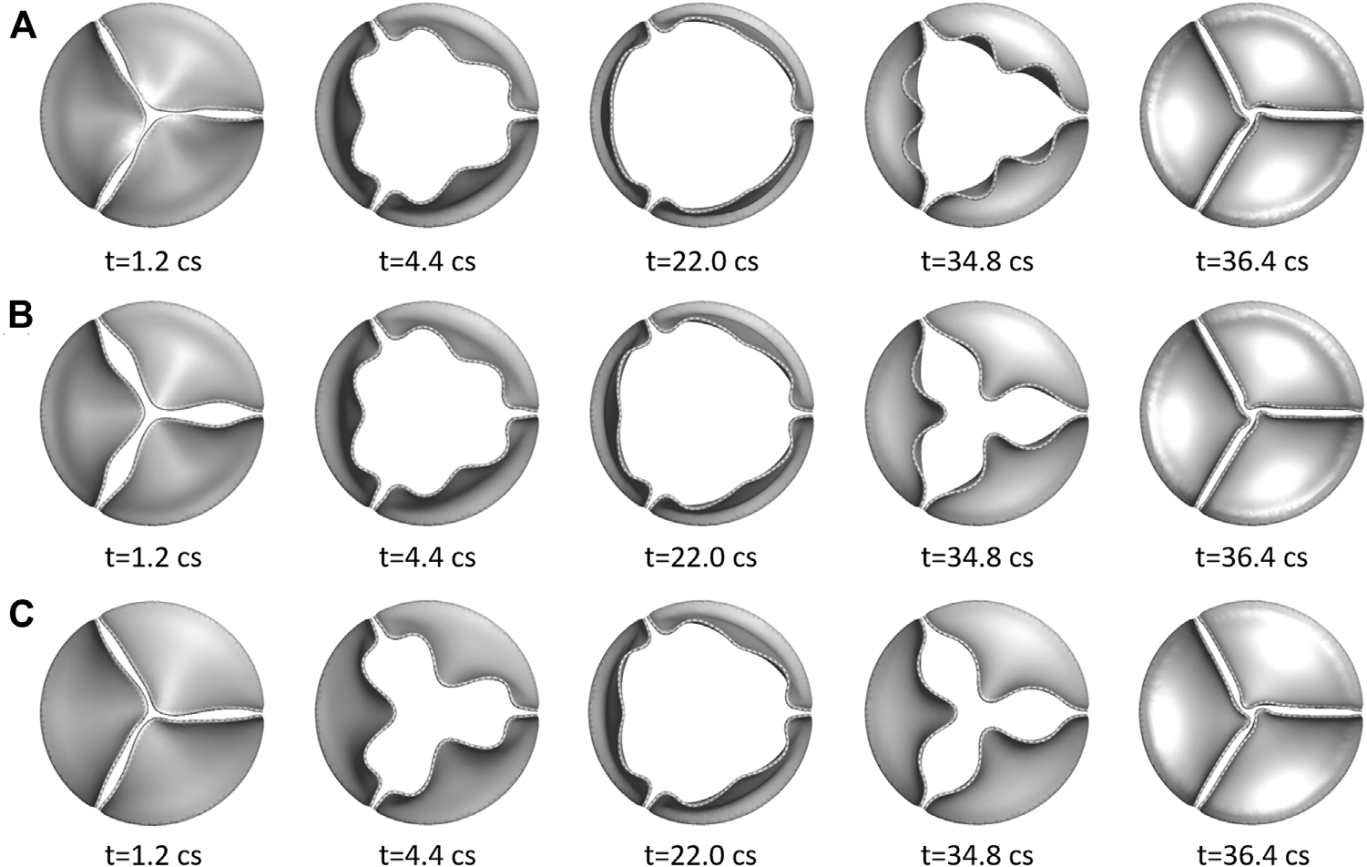
Three rows show the opening and closing phases of the three aortic valves at multiple time instants: **(A)** E = 375 kPa; **(B)** E = 750 kPa; and **(C)** E = 1500 kPa.

Figure 5 shows the opening and closing phases of the aortic valves for all three cases. The opening process for the more compliant aortic valve ( E=375 and 750  kPa) is faster than its stiffer counterpart E=1500 kPa (see t=4.4 cs). At peak systole t=22.0 cs, all three aortic valves reached the fully open stage. The closing phase of the more compliant leaflet valve is slightly delayed (see t=34.8 cs), which can also be seen from the GOA history shown in Figure 4B.

### Hemodynamic Forces and Stresses

**FIGURE 6:**
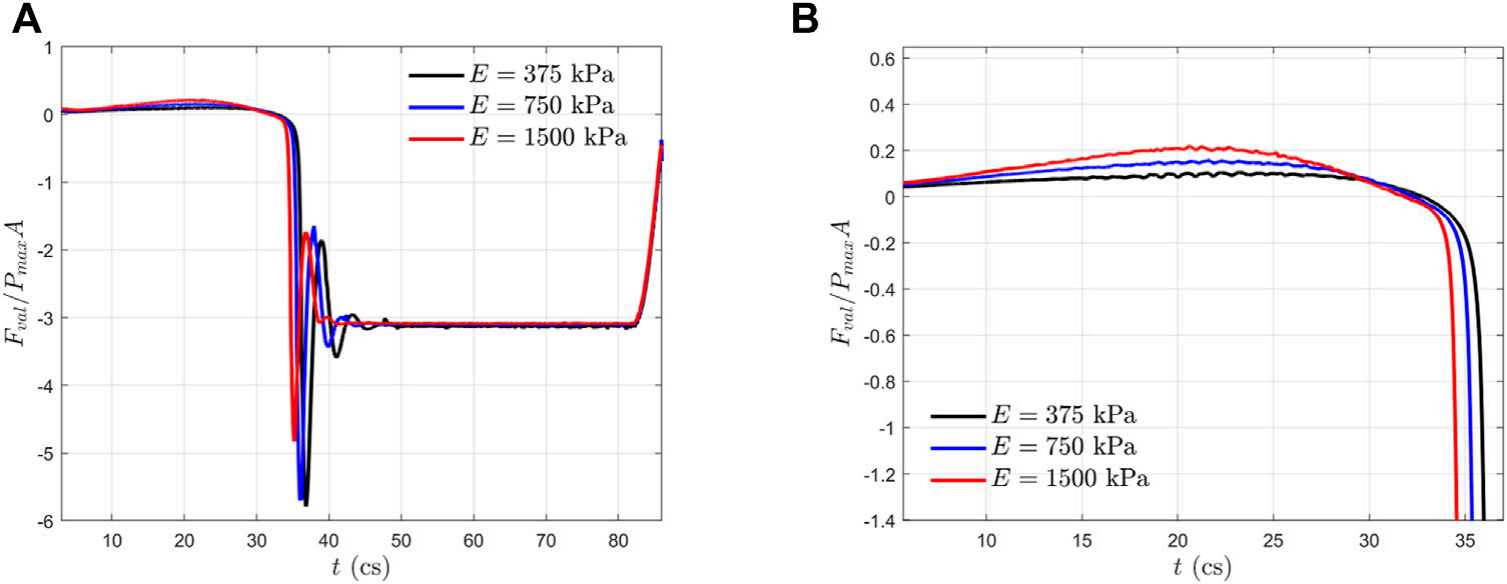
**(A)** Normalized axial hemodynamic forces experienced by the aortic valves within one cardiac cycle; and **(B)** a zoom-in view of **(A)** during the systole phase.

In Figure 6, we plotted the normalized hemodynamic force along the *x*-direction experienced by the aortic valve. During the systolic phase, the more compliant aortic leaflet valve ( E=375 kPa) experienced a lower axial hemodynamic force when compared with its stiffer counterpart. The momentum balance study presented in our previous work (Chen and Luo, 2018; Chen and Luo, 2020) has revealed the following approximation:

$$\text{Δ}PA\approx \;\rho L\dot{Q}+F_{val}$$ (6)

where ΔP is the transvalvular pressure drop shown in Figure 2, $A=\pi D^{2}/4$ is the cross-section area, $\dot{Q}=dQ/dt$ is the time derivative of the flow rate, and $F_{val}$ is the total axial hemodynamic force on the leaflet surfaces (both aortic and ventricular sides included). Given the pressure loading ΔPA , the smaller hemodynamic force $F_{val}$ of the more compliant aortic valve in systole corresponds to a greater acceleration of the fluid column $\rho L\dot{Q}$ inside the aortic tube, which resulted in a higher transient flow rate Q  for the higher compliance aortic valves, as presented in Figure 4A. This result means that the more compliant aortic leaflet valve takes more advantage of the transvalvular pressure drop for blood delivery. Meanwhile, the dip of negative hemodynamic force during the rapid closure is larger for a higher compliant valve to stop the reversal of flow. The magnitudes of the forces are −5.8 , −5.7  , and −4.8 for E=375 , 750  , and 1500 kPa, respectively.

**FIGURE 7:**
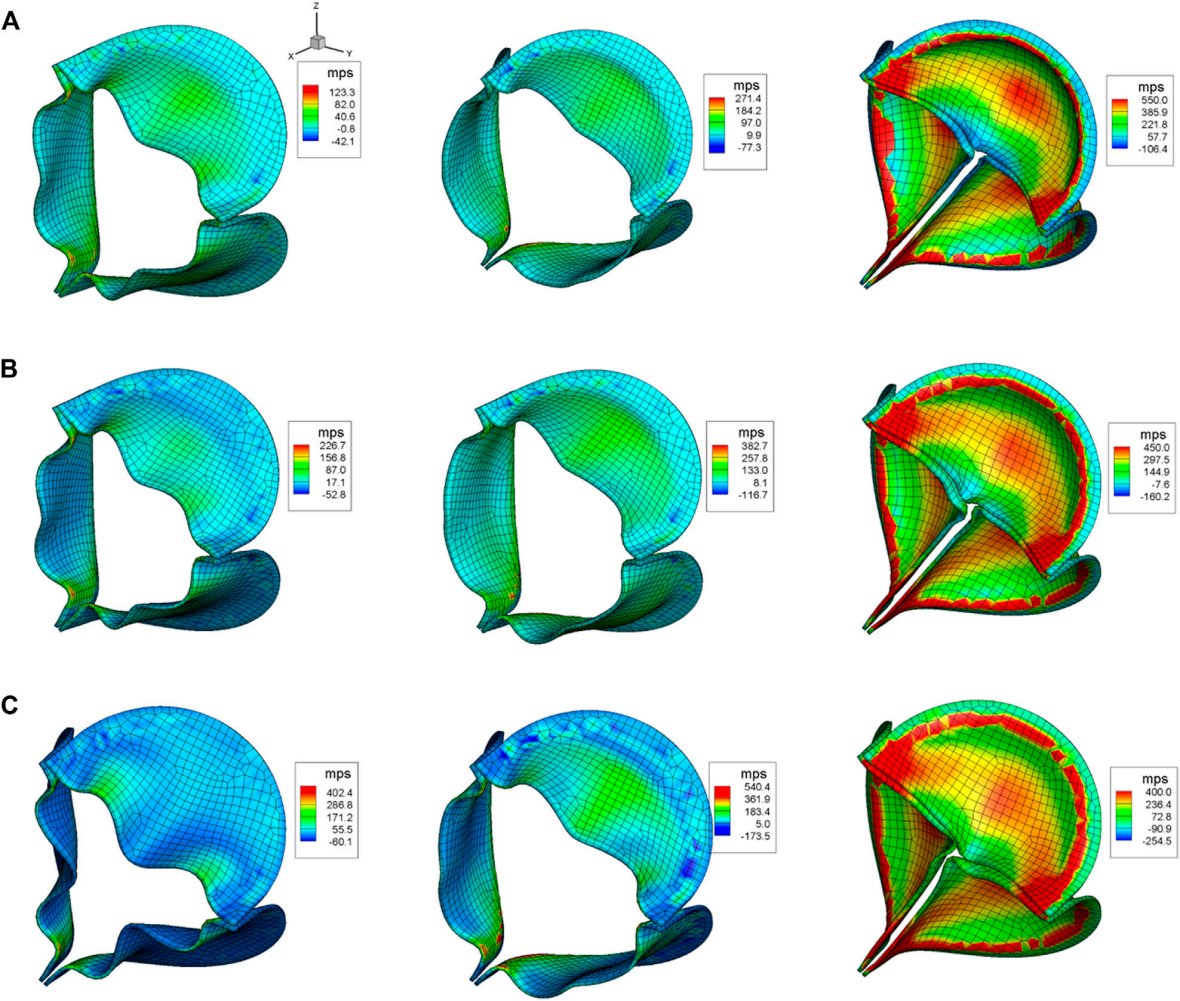
Aortic leaflet stresses for **(A)** E = 375 kPa; **(B)** E = 750 kPa; and **(C)** E = 1500 kPa during the opening and closing phases at t = 4.4, 22.0, and 34.8 cs.

Figure 7 shows the maximum principal stress (MPS) of the aortic leaflet for all the three cases during the opening phase ( t=4.4 cs), at peak systole ( t=22.0 cs), and at the instant of peak “water-hammer” which corresponds to the maximum negative hemodynamic impact shown in Figure 6A. The stress distributions on the leaflet surfaces are qualitatively similar among the three valves, while their magnitudes are significantly different. The leaflet stress for the more compliant aortic valve is substantially lower during both the opening and peak systole phases (the first two columns in Figure 7 can be referred). For example, at peak systole, the maximum value of stress is around 271 kPa for E=375 kPa, while it increases to 383 and 540 kPa for E=750 and 1500 kPa, respectively. The “water-hammer” effect emerges because of the aortic valve’s rapid shutdown and the ensuing impingement of fast-stopped blood on the leaflets, which creates high stress on the aortic side of the leaflets (the third column of Figure 7 can be referred). In contrast to the systole phase, at the instant of peak “water-hammer”, the strength of “water-hammer” is stronger for the more compliant valve, which produces a larger hemodynamic force on the valve, and the leaflet stress for the more compliant valve is higher than its stiffer counterparts. The maximum value of stress is about 550 kPa for E=375 kPa, and it reduces to about 450 and 400 kPa for E=750 and 1500  kPa in the belly region, respectively. In addition, the more compliant aortic leaflet valve is also found to be pushed more upstream toward the inlet due to this strong impact, which also generates higher stress along the fixed attachment.

## Discussions

All the three valves in this study have shown proper opening and closing dynamics. However, our results show that the more compliant aortic valves with smaller Young’s modulus E have a higher cardiac output, larger GOA, and lower hemodynamic resistance. Overall, the aortic leaflet stresses are substantially lower in the belly region within the more compliant aortic leaflet valve tissue, which may mitigate leaflet fatigue and potential calcification.

### Mechanical Stress and Calcification

In the present study, we simulated the stress distribution of the leaflets made of bovine pulmonary visceral pleura (bPVP). The bPVP is the layer of the serous membrane overlying the lungs and is composed of abundant collagen and elastin. Due to the larger proportion of elastin, PVP is more elastic and resilient when compared with porcine heart valves and bovine pericardium, which may render bPVP potentially advantageous for mitigation of calcification due to its much smaller Young’s modulus than that of porcine heart valves and bovine pericardium, which are collagen-dominant biomaterials and have been broadly utilized to construct bioprosthetic heart valves (BHVs) (Lindberg and Badylak, 2001; Maestro et al., 2006; Manji et al., 2012; Gauvin et al., 2013; Kheradvar et al., 2015; Dasi et al., 2017). Our simulation demonstrates that the mechanical stresses in bPVP leaflets are much smaller than those in the biomaterial where the Young’s modulus is similar to that of the bovine pericardium during the systole, even though some increase is also found during the valves’ rapid close and the ensuing “water-hammer” effect. The overall smaller mechanical stresses in bPVP leaflets may mitigate the degradation and calcification of leaflets in the bPVP prosthetic valve. This hypothesis remains to be validated in preclinical large animal models and clinical studies.

### The Relationship Between Aortic Valve Performance and Bending Rigidity

The aortic valve performance is determined by both the blood flow and valve structure since the instantaneous leaflet shape and motion are a result of the two-way interaction between them. In our previous work (Chen and Luo, 2020), a dimensionless parameter named normalized bending rigidity $E_{B}^{*}=E_{B}/\left(\Delta pR^{4}/L\right)$ is defined with an optimal range for proper valve opening performance. $E_{B}=Eh^{3}/12\left(1-\nu^{2}\right)$ is the bending rigidity, R is the aorta radius, and Δp/L is the pressure gradient applied to drive the blood flow through the aortic valve. Given the driven pressure gradient Δp/L , Young’s modulus E , and Poisson’s ratio ν , the optimal range of $E_{B}^{*}\in \left[0.003,\;0.4\right]$ is determined by investigating the aortic valve performance with leaflet thickness h varying from 0.005 to 0.8  mm. In the current work, the leaflet thickness is fixed with h=0.03 mm, while Young’s modulus E is varied from 375 and 750 to 1500 kPa to represent the material property difference between the more compliant PVP and its stiffer counterpart of porcine/bovine pericardium. Following the definition of $E_{B}^{*}$ proposed in Ref. 16, we obtained $E_{B}^{*}=8\times 10^{-3}$ , $1.6\times 10^{-2}$ and $3.1\times 10^{-2}$ for E = 375, 750, and 1500 kPa, respectively. We thus observed that for all the three cases studied in this work, their $E_{B}^{*}$ fall within the optimal range of $E_{B}^{*}$ between 0.003 and 0.04 . Thus, the three aortic valves in this work can perform properly without experiencing either evident leaflet fluttering ( $E_{B}^{*}$ too small) or opening difficulty ( $E_{B}^{*}$ too large). This is also confirmed by our results on the flow rate Q , valve opening area GOA, and leaflet deformation in the previous section.

### Relationship Between the Aortic Leaflet Stress and Bending Rigidity

In our previous work (Chen and Luo, 2020), the optimal range of $E_{B}^{*}$ is established based on the proper valve opening area (GOA). Excessively small and large $E_{B}^{*}\notin \left[0.003,\;\;0.4\right]$ are excluded to prevent severe leaflet fluttering and valve opening difficulty. In this work, we extended our previous study by providing an additional dimension of investigating the relationship between the aortic leaflet stress and normalized bending rigidity $E_{B}^{*}\;$ within the optimal range. This dimension is important since a recent FE study has pointed out that the significantly elevated mechanical stress is strongly correlated with the regions with a high risk of calcium buildup (Qin et al., 2020). Even though $E_{B}^{*}$ for all three cases in this work fall within the optimal range $E_{B}^{*}\notin \left[0.003,\;\;0.4\right]$ , the difference in the magnitudes of the aortic leaflet stress (maximum principal stress) is evident (as shown in Figure 7). During the systole phase, the stress is substantially lower for the more compliant leaflet valve, which suggests that PVP could serve as a potentially advantageous biomaterial over traditional pericardium for the construction of BHVs in the systole period. However, during the fast-closing phase, the more compliant aortic valve experiences a stronger “water-hammer” effect with a high peak hemodynamic force impinging on the leaflets, which pushes the aortic valve more toward the left ventricle side and results in an increase of stress. Thus, the potential advantage of PVP might be compromised due to the “water-hammer” effect during the fast-closing phase. Future studies will be conducted to investigate the performance of PVP in our follow-up effort.

## Conclusion

Porcine heart valves and bovine pericardium are collagen-based with relatively minor elastin, while the lung tissue, PVP, is abundant in both collagen and elastin. PVP has some of the merits of porcine heart valves and bovine pericardium but adds substantial elasticity due to the larger proportion of elastin. To take the additional elasticity effect into account, we considered three cases of Young’s modulus, E=375 , 750 , and 1500 kPa, in this computational study. The corresponding normalized bending rigidity $E_{B}^{*}=8\times 10^{-3}$ , $1.6\times 10^{-2}$ , and $3.1\times 10^{-2}$ , respectively, where all fall within the optimal range for a proper aortic valve performance, as proposed in our previous work (Chen and Luo, 2020). Our results show that the more compliant leaflet valve ( E=375 kPa) can generate a higher stroke volume, larger opening area (GOA), and proper leaflet deformation without incurring significant leaflet fluttering during the systole phase. In particular, the aortic leaflet stresses in the belly region are substantially lower for the more compliant aortic leaflet valves during the systole phase, which may reduce the risk of calcium buildup observed in the high-stress region and hence suggests the potentially advantageous application of PVP biomaterial for the construction of BHVs. This hypothesis-generating study highlights the need for future clinical studies to test the hypothesis that the PVP-based valve leaflets will have lower degradation or calcification rates.

## Data Availability

The original contributions presented in the study are included in the article/Supplementary Material, further inquiries can be directed to the corresponding author.

## References

[B1] Borazjani I. (2013). Fluid-structure Interaction, Immersed Boundary-Finite Element Method Simulations of Bio-Prosthetic Heart Valves. Computer Methods Appl. Mech. Eng. 257, 103–116. 10.1016/j.cma.2013.01.010

[B2] Chen Y. Luo H. (2018). A Computational Study of the Three-Dimensional Fluid-Structure Interaction of Aortic Valve. J. Fluids Structures 80, 332–349. 10.1016/j.jfluidstructs.2018.04.009

[B3] Chen Y. Luo H. (2020). Pressure Distribution over the Leaflets and Effect of Bending Stiffness on Fluid–Structure Interaction of the Aortic Valve. J. Fluid Mech. 883. 10.1017/jfm.2019.904

[B4] Dasi L. P. Hatoum H. Kheradvar A. Zareian R. Alavi S. H. Sun W. (2017). On the Mechanics of Transcatheter Aortic Valve Replacement. Ann. Biomed. Eng. 45 (2), 310–331. 10.1007/s10439-016-1759-3 27873034 PMC5300937

[B5] Ferrans V. J. Spray T. L. Billingham M. E. Roberts W. C. (1978). Structural Changes in Glutaraldehyde-Treated Porcine Heterografts Used as Substitute Cardiac Valves. Am. J. Cardiol. 41, 1159–1184. 10.1016/0002-9149(78)90873-1 96684

[B6] Gauvin R. Marinov G. Mehri Y. Klein J. Li B. Larouche D. (2013). A Comparative Study of Bovine and Porcine Pericardium to Highlight Their Potential Advantages to Manufacture Percutaneous Cardiovascular Implants. J. Biomater. Appl. 28, 552–565. 10.1177/0885328212465482 23142967

[B7] Gilmanov A. Barker A. Stolarski H. Sotiropoulos F. (2019). Image-guided Fluid-Structure Interaction Simulation of Transvalvular Hemodynamics: Quantifying the Effects of Varying Aortic Valve Leaflet Thickness. Fluids 43, 119. 10.3390/fluids4030119

[B8] Gilmanov A. Le T. B. Sotiropoulos F. (2015). A Numerical Approach for Simulating Fluid Structure Interaction of Flexible Thin Shells Undergoing Arbitrarily Large Deformations in Complex Domains. J. Comput. Phys. 300, 814–843. 10.1016/j.jcp.2015.08.008

[B9] Gilmanov A. Sotiropoulos F. (2016). Comparative Hemodynamics in an Aorta with Bicuspid and Trileaflet Valves. Theor. Comput. Fluid Dyn. 30, 67–85. 10.1007/s00162-015-0364-7

[B10] Griffith B. E. (2012). Immersed Boundary Model of Aortic Heart Valve Dynamics with Physiological Driving and Loading Conditions. Int. J. Numer. Meth. Biomed. Engng. 28 (3), 317–345. 10.1002/cnm.1445 25830200

[B11] Griffith B. E. Luo X. McQUEEN D. M. Peskin C. S. (2009). Simulating the Fluid Dynamics of Natural and Prosthetic Heart Valves Using the Immersed Boundary Method. Int. J. Appl. Mech. 01 (01), 137–177. 10.1142/s1758825109000113

[B12] Hole J. W. (1996). Hole’s Human Anatomy & Physiology. 7th ed. Dubuque: Wm. C. Brown Publishers.

[B13] Hsu M.-C. Kamensky D. Xu F. Kiendl J. Wang C. Wu M. C. H. (2015). Dynamic and Fluid-Structure Interaction Simulations of Bioprosthetic Heart Valves Using Parametric Design with T-Splines and Fung-type Material Models. Comput. Mech. 55 (6), 1211–1225. 10.1007/s00466-015-1166-x 26392645 PMC4574293

[B14] Kamensky D. Hsu M.-C. Schillinger D. Evans J. A. Aggarwal A. Bazilevs Y. (2015). An Immersogeometric Variational Framework for Fluid-Structure Interaction: Application to Bioprosthetic Heart Valves. Computer Methods Appl. Mech. Eng. 284, 1005–1053. 10.1016/j.cma.2014.10.040 PMC4274080 25541566

[B15] Kheradvar A. Groves E. M. Goergen C. J. Alavi S. H. Tranquillo R. Simmons C. A. (2015). Emerging Trends in Heart Valve Engineering: Part II. Novel and Standard Technologies for Aortic Valve Replacement. Ann. Biomed. Eng. 43 (4), 844–857. 10.1007/s10439-014-1191-5 25449148

[B16] Kim H. Lu J. Sacks M. S. Chandran K. B. (2008). Dynamic Simulation of Bioprosthetic Heart Valves Using a Stress Resultant Shell Model. Ann. Biomed. Eng. 362, 262–275. 10.1007/s10439-007-9409-4 18046648

[B17] Kuang H. Xuan Y. Lu M. Mookhoek A. Wisneski A. D. Guccione J. M. (2017). Leaflet Mechanical Properties of Carpentier-Edwards Perimount Magna Pericardial Aortic Bioprostheses. J. Heart Valve Dis. 261, 81–89. PMC8505361 28544835

[B18] Lindberg K. Badylak S. F. (2001). Porcine Small Intestinal Submucosa (SIS): a Bioscaffold Supporting *In Vitro* Primary Human Epidermal Cell Differentiation and Synthesis of Basement Membrane Proteins. Burns 27 (3), 254–266. 10.1016/s0305-4179(00)00113-3 11311519

[B19] Lu X. Han L. Golts E. Baradarian S. Kassab G. S. (2020). Homologous and Heterologous Assessment of a Novel Biomaterial for Venous Patch. J. Vasc. Surg. Venous Lymphatic Disord. 83, 458–469. 10.1016/j.jvsv.2019.09.011 31837973

[B20] Luo H. Dai H. Ferreira de Sousa P. J. S. A. Yin B. (2012). On the Numerical Oscillation of the Direct-Forcing Immersed-Boundary Method for Moving Boundaries. Comput. Fluids 56, 61–76. 10.1016/j.compfluid.2011.11.015

[B21] Maestro M. M. Turnay J. Olmo N. Fernández P. Suárez D. Páez J. M. G. (2006). Biochemical and Mechanical Behavior of Ostrich Pericardium as a New Biomaterial. Acta Biomater. 2, 213–219. 10.1016/j.actbio.2005.11.004 16701880

[B22] Manji R. A. Menkis A. H. Ekser B. Cooper D. K. (2012). The Future of Bioprosthetic Heart Valves. Indian J. Med. Res. 135 (2), 150–151. 22446853 PMC3336842

[B23] Mao W. Li K. Sun W. (2016). Fluid-Structure Interaction Study of Transcatheter Aortic Valve Dynamics Using Smoothed Particle Hydrodynamics. Cardiovasc. Eng. Tech. 7 (4), 374–388. 10.1007/s13239-016-0285-7 PMC5289304 27844463

[B24] Marom G. Peleg M. Halevi R. Rosenfeld M. Raanani E. Hamdan A. (2013). Fluid-structure Interaction Model of Aortic Valve with Porcine-specific Collagen Fiber Alignment in the Cusps. J. Biomech. Eng. 135 (10), 101001–101006. 10.1115/1.4024824 23775457

[B25] Oveissi F. Naficy S. Lee A. Winlaw D. S. Dehghani F. (2020). Materials and Manufacturing Perspectives in Engineering Heart Valves: a Review. Mater. Today Bio 5, 100038. 10.1016/j.mtbio.2019.100038 PMC7083765 32211604

[B26] Qin T. Caballero A. Mao W. Barrett B. Kamioka N. Lerakis S. (2020). The Role of Stress Concentration in Calcified Bicuspid Aortic Valve. J. R. Soc. Interf. 17167, 20190893. 10.1098/rsif.2019.0893 PMC7328384 32517630

[B27] Reul H. Vahlbruch A. Giersiepen M. Schmitz-Rode T. Hirtz V. Effert S. (1990). The Geometry of the Aortic Root in Health, at Valve Disease and after Valve Replacement. J. Biomech. 23 (2), 181–191. 10.1016/0021-9290(90)90351-3 2312522

[B28] Robicsek F. Thubrikar M. J. (2002). Mechanical Stress as Cause of Aortic Valve Disease Presentation of a New Aortic Root Prosthesis. Acta Chirurgica Belgica 102 (1), 1–6. 10.1080/00015458.2002.11679253 11925731

[B29] Schoen F. J. Levy R. J. (2005). Calcification of Tissue Heart Valve Substitutes: Progress toward Understanding and Prevention. Ann. Thorac. Surg. 79 (3), 1072–1080. 10.1016/j.athoracsur.2004.06.033 15734452

[B30] Singh R. Strom J. A. Ondrovic L. Joseph B. VanAuker M. D. (2008). Age-related Changes in the Aortic Valve Affect Leaflet Stress Distributions: Implications for Aortic Valve Degeneration. J. Heart Valve Dis. 17 (3), 290–299. 18592926

[B31] Soltany Sadrabadi M. Hedayat M. Borazjani I. Arzani A. (2021). Fluid-structure Coupled Biotransport Processes in Aortic Valve Disease. J. Biomech. 117 (2021), 110239. 10.1016/j.jbiomech.2021.110239 33515904

[B32] Swanson W. M. Clark R. E. (1974). Dimensions and Geometric Relationships of the Human Aortic Value as a Function of Pressure. Circ. Res. 35 (6), 871–882. 10.1161/01.res.35.6.871 4471354

[B33] Thubrikar M. J. Deck J. D. Aouad J. Nolan S. P. (1983). Role of Mechanical Stress in Calcification of Aortic Bioprosthetic Valves. J. Thorac. Cardiovasc. Surg. 86 (1), 115–125. 10.1016/s0022-5223(19)39217-7 6865456

[B34] Tian F.-B. Dai H. Luo H. Doyle J. F. Rousseau B. (2014). Fluid-structure Interaction Involving Large Deformations: 3D Simulations and Applications to Biological Systems. J. Comput. Phys. 258, 451–469. 10.1016/j.jcp.2013.10.047 PMC3884079 24415796

[B35] Weinberg E. J. Kaazempur-Mofrad M. R. (2005). On the Constitutive Models for Heart Valve Leaflet Mechanics. Cardiovasc. Eng. 5 (1), 37–43. 10.1007/s10558-005-3072-x

